# Cellular Reprogramming, Genome Editing, and Alternative CRISPR Cas9 Technologies for Precise Gene Therapy of Duchenne Muscular Dystrophy

**DOI:** 10.1155/2017/8765154

**Published:** 2017-05-15

**Authors:** Peter Gee, Huaigeng Xu, Akitsu Hotta

**Affiliations:** ^1^Center for iPS Cell Research and Application (CiRA), Kyoto University, 53 Kawahara-cho, Shogoin, Sakyo-ku, Kyoto 606-8507, Japan; ^2^Institute for Integrated Cell Material Sciences (iCeMS), Kyoto University, Yoshida Ushinomiya-cho, Sakyo-ku, Kyoto 606-8507, Japan

## Abstract

In the past decade, the development of two innovative technologies, namely, induced pluripotent stem cells (iPSCs) and the CRISPR Cas9 system, has enabled researchers to model diseases derived from patient cells and precisely edit DNA sequences of interest, respectively. In particular, Duchenne muscular dystrophy (DMD) has been an exemplary monogenic disease model for combining these technologies to demonstrate that genome editing can correct genetic mutations in DMD patient-derived iPSCs. DMD is an X-linked genetic disorder caused by mutations that disrupt the open reading frame of the *dystrophin* gene, which plays a critical role in stabilizing muscle cells during contraction and relaxation. The CRISPR Cas9 system has been shown to be capable of targeting the *dystrophin* gene and rescuing its expression in in vitro patient-derived iPSCs and in vivo DMD mouse models. In this review, we highlight recent advances made using the CRISPR Cas9 system to correct genetic mutations and discuss how emerging CRISPR technologies and iPSCs in a combined platform can play a role in bringing a therapy for DMD closer to the clinic.

## 1. Induced Pluripotent Stem Cells for Disease Modeling

Induced pluripotent stem cells (iPSCs), first established by Takahashi et al. in 2006, are invaluable tools for disease modeling that could one day provide a source of healthy autologous cells needed for regenerative medicine applications [1, 2]. Induced by reprogramming somatic cells, such as skin or blood, with four key transcription factors (OCT3/4, SOX2, KL4, and c-MYC), human pluripotent stem cells with similar features to embryonic stem cells can be obtained. These cells possess several advantageous features including unlimited self renewal in culture and the ability to be differentiated into endoderm, ectoderm, and mesoderm, including pancreatic *β*-cells, cardiomyocytes, neurons, and myocytes [2]. Moreover, iPSCs are particularly useful for studying the underlying mechanism of genetic disorders because they can recapitulate patient genotypes and cellular phenotypes upon differentiation, thus, can be used for screening chemical libraries to identify novel therapies in disease relevant cells [3]. In fact, initiatives to establish a large number of patient-derived iPSCs in the USA, EU, UK [4], and Japan [5] are ongoing, demonstrating the importance of iPSCs for disease modeling and new drug development.

While patient-derived iPSCs have been established for many diseases (e.g., FTLD-Tau [6], type I diabetes [7], Down syndrome, Parkinson's disease [8], etc.), we focus on the utility of those from Duchenne muscular dystrophy (DMD) patients [8–11]. Many DMD iPSC lines have been established containing different types of mutations in the *dystrophin* gene ranging from nonsense mutations to whole exon deletions or duplications (Table 1).

## 2. What Is Duchenne Muscular Dystrophy and What Treatments Are Currently Available?

DMD is an X-linked genetic muscle wasting disease that occurs in approximately 1 in 3500 males and affects mainly skeletal and cardiac muscles [16]. DMD patients are born apparently normal, but start to display symptoms of delayed muscle development during childhood, lose their walking ability after 10 years of age, become dependent on aspirators in their 20s, and generally do not survive past the age of late 30s, eventually succumbing to cardiac or respiratory failure [17]. The main cause of the disease is attributed to mutations in one of the largest protein-coding genes in the human genome called dystrophin that spans 79 exons (2.2 megabases) and stabilizes muscle cells by anchoring the cytoskeleton to the extracellular matrix with other proteins, in a complex known as the dystrophin-associated glycoprotein complex (DAGC) [18]. Dp427m is the main muscle isoform of dystrophin protein, which has an mRNA length of 14 kb and protein molecular weight of 427 kDa. Importantly, the N- and C-terminal regions of the protein are critical for functional anchoring of dystrophin to actin and DAGC while the central rod domain, consisting of 24 triple helix rod domains and 4 hinge regions, connects both ends and appears to be expendable to a certain extent [19]. The majority of genetic mutations in the *dystrophin* gene found in DMD patients are large deletions that disrupt the ORF of the dystrophin protein, which are mainly found in frontal exons 2–20 or a deletion hot spot exons 45–55, accounting for over 60% of mutations in DMD patients [20]. On the other hand, in-frame mutations in the *dystrophin* gene are known to cause a milder disease phenotype, named Becker muscular dystrophy (BMD) [21]. BMD patients show a broad spectrum of disease phenotypes, but in general, retain their ability to walk and live longer than DMD patients. In BMD patients, in-frame deletions in the *dystrophin* gene lead to the translation of truncated but functional dystrophin protein. In fact, BMD patients who lack more than half of the dystrophin protein coding sequence in the rod domain have been documented [21, 22]. The existence of these BMD patients offers evidence that expression of a truncated dystrophin protein serves as a basis to treat DMD patients with smaller dystrophin protein variants.

Skeletal muscle cells missing functional dystrophin protein are susceptible to membrane permeability and leakage of Ca^2+^ ions after muscle contraction and relaxation, ultimately resulting in cell death. Over time, muscle cells are replaced by fat and scar tissue. Typical therapies for DMD include corticosteroid treatments that can delay a wheelchair-bound state; however, caution is needed for adverse effects that include behavioral changes, fractures, cataracts, weight gain, and cushingoid appearance [23]. Moreover, current therapeutic options to treat the root cause of the disease itself are limited.

To this end, several therapies have been explored in clinical trials to treat the cause of DMD. In one clinical trial, gene therapy was used to supply minidystrophin [24], a 60% truncated form of dystrophin cDNA lacking a large portion of the rod domain and a C-terminal domain [25], delivered through a viral vector. Unfortunately, viral-mediated delivery of microdystrophin into patients failed to establish sustained protein expression, possibly due to an immune response [24]. Another therapy used antisense oligonucleotides (AONs) that are able to induce exon skipping at the pre-mRNA level for rescuing truncated dystrophin protein expression. Recently, the FDA conditionally approved Eteplirsen (Exondy51) [26], an AON that skips exon 51, but its half-life in blood is only 3 hours, hence a weekly injection is required. It is still unclear whether AONs will be able to provide long-term benefits in preventing muscular dystrophy in patients.

## 3. Considerations for Allogeneic or Autologous iPS Cell Therapy to Treat DMD Going Forward

As DMD patients suffer from severe muscle atrophy, cell transplantation therapy would be a rational approach. Obtaining functional myoblast progenitor cells from autologous or human leukocyte antigen- (HLA-) matched allogenic iPSCs has advantages for expansion and potential clinical use over other methods such as direct isolation of primary myoblasts and myoblasts derived from reprogrammed fibroblasts. Primary myoblasts are generally immortalized by oncogenic factors including the SV40 large T antigen and telomerase reverse transcriptase (*TERT*), making their use for transplantation unreasonable. Reprogrammed myoblasts from fibroblasts by *MYOD1* [27] also suffer from the same problem as primary myoblasts because they require immortalization for long-term survival and expansion [28]. On the other hand, iPSCs can be maintained indefinitely and then converted into myoblast progenitors [29]. This is particularly important because clinical trials where allogeneic myoblasts were transplanted into DMD patients revealed that low cell survival, poor cell migration, and potential immune clearance are issues, which means a high cell number is needed [30]. To further improve cell transplantation, identification of an appropriate cell type with better survival and engraftment should be determined. For preparation of large cell numbers, the selected differentiation protocol from iPSCs needs to be robust and scalable.

To minimize immunogenic reactions, autologous human iPSCs could be edited ex vivo and then transplanted back into the patient. The autologous approach could work similarly to a mouse study in which fibroblasts from a severe DMD mouse model (*mdx*) that lacks both *utrophin* and *dystrophin* genes were reprogrammed into iPSCs ex vivo and transduced with a *Sleeping Beauty* transposon to express microdystrophin cDNA [31]. After differentiation into myogenic progenitor cells, the cells were transplanted back into the *mdx* mice by engraftment or systemic delivery [31]. Both led to dystrophin protein expressing skeletal muscle cells, improved muscle strength, and, importantly, the establishment of satellite muscle cells for a continual supply of corrected skeletal muscle cells. In the human context, iPSCs could be established and then differentiated into myoblast progenitor cells or muscle stem cells for transplantation back into the patient. As DMD iPSCs carry the same genetic mutation as the original patient, functional dystrophin protein must be restored before the transplantation. The classical approach would be to transduce cDNA by a vector, such as a human artificial chromosome [11] or *Sleeping Beauty* transposon vector [31]. More recently, genome editing approaches have been evolving to correct the *Dystrophin* mutation(s).

## 4. How Does Genome Editing Work?

Genome editing can be used to facilitate DNA repair after a double stranded DNA break (DSB) is induced by a programmable nuclease [32]. Thereafter, two predominant DNA repair pathways are induced. One involves homologous recombination (HR), which requires the presence of a DNA template with homology regions overlapping each side of the cleaved DNA to be precisely inserted into the DSB site [33]. The other DNA repair pathway is the predominant one, called NHEJ (nonhomologous end joining), and leads to insertions or deletions (indels) being introduced to patch up the DSB site. NHEJ is more frequent and has been estimated to occur within 30 minutes as opposed to HR, which takes as long as 7 hours [34]. Both of these approaches have been utilized in combination with DNA nucleases for genome editing purposes.

## 5. CRISPR Cas9 Nucleases

Nucleases available for gene editing such as meganucleases, TALENs (transcription activator-like effector nucleases), and ZFNs (zinc-finger nucleases) rely on engineering the DNA binding domain for recognizing specific DNA sequences (reviewed in [35, 36]). In contrast, CRISPR (clustered regularly interspaced short palindromic repeats) Cas9 (CRISPR associated protein) uses complementary guide RNA, hence it is highly versatile and has become the preferred nuclease of choice in the genome editing field, enabling scientists to quickly establish disease models, create gene knockouts for studying cellular phenotypes, and model gene correction for monogenic diseases.

CRISPR-Cas9 was first identified as an adaptive immune system in bacteria against invading bacteriophages and later harnessed into a tool for DNA editing [37]. It was quickly adapted for use in mammalian cells and has also been proven to function in many organisms. The Type II CRISPR system consists of a nuclease called Cas9 and a single guide RNA molecule (sgRNA), which is a fusion of two RNA components, transactivating RNA (tracrRNA) and CRISPR RNA. Cas9 complexes with sgRNA and is guided to a targeted DNA sequence by a programmable 20 bp sequence that lies at the most 5′-terminal portion of the sgRNA. Importantly, the 20 bp targeting sequence must reside next to a defined protospacer adjacent motif (PAM) that varies in sequence and length depending on the Cas9 nuclease being used for DNA cleavage. In this respect, there are a variety of Cas9 nucleases to choose from depending on one's targeting needs.

CRISPR Cas9 from *Streptococcus pyogenes* (SpCas9) is the most commonly utilized for DNA editing because it is extensively characterized in respect to its structure and activity [37–40]. SpCas9 cDNA is approximately 4.1 kb long and translates into a 1368 amino acid protein [37]. Owing to the fact that its PAM sequence (NGG) is relatively common in the human genome, it offers flexibility for designing sgRNA against DNA target sequences. The Cas9 cDNA from *Staphylococcus aureus* (SaCas9) is smaller than that from SpCas9 by nearly 1000 bp and encodes a 1053 amino acid protein. SaCas9 has a “NGGRRT” PAM requirement; therefore, its targetable density is lower than that of SpCas9 [41].

In combination with iPSCs, genetic mutations in patient-derived iPSCs have been successfully corrected by CRISPR-Cas9 system for several diseases, such as *β*-thalassemia [42], Niemann-Pick disease Type C [43], hemophilia A [44], and DMD [12, 13, 45]. Both Sp- and SaCas9 have been used successfully to correct dystrophin gene mutations in *mdx* mice and patient-derived myoblasts and iPSCs.

## 6. How Can CRISPR Cas9 Be Applied to DMD?

**Figure 1:**
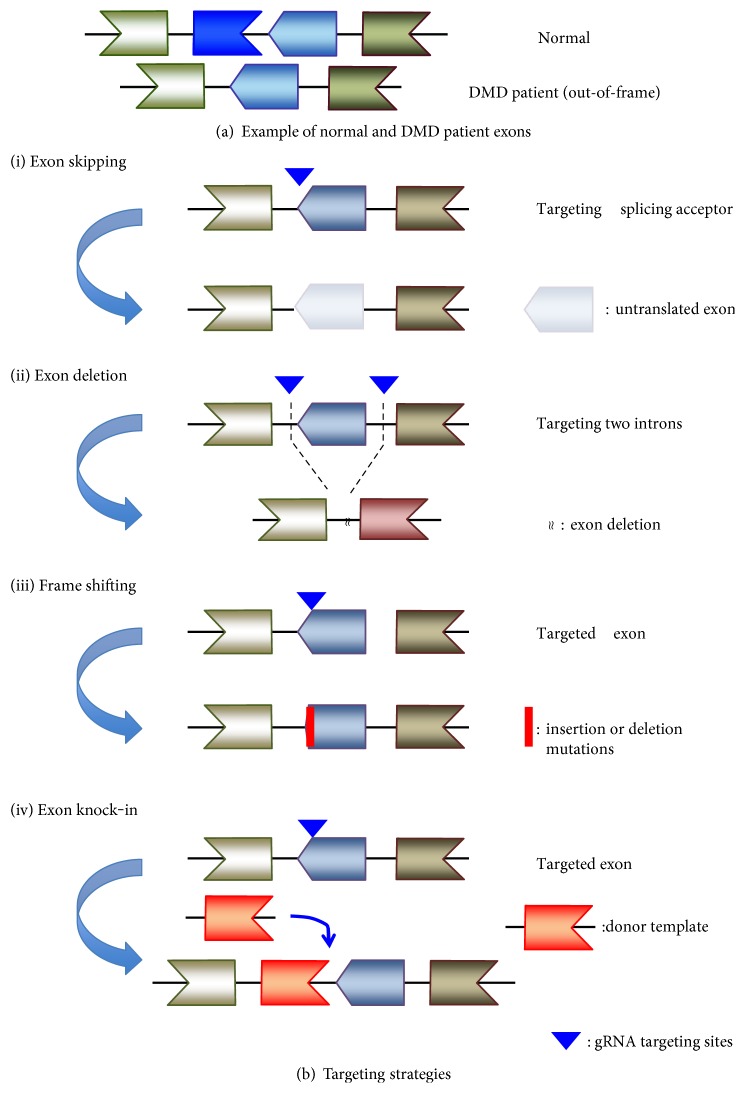
*Dystrophin* gene targeting strategies by CRISPR Cas9. (a) Examples of normal and patient *dystrophin* gene exons. Individual exons are represented by beige, dark blue, light blue, and brown. In healthy patient genomes, the exons are in frame and will lead to the expression of a full protein. In DMD patient genomes, the deletion of the dark blue exon leads to a frame shift, disrupting the ORF and causing a premature stop codon. (b) Four main strategies of genome editing to correct the ORF of the *dystrophin* gene: (i) for exon skipping, sgRNA is designed to target a splicing acceptor. This disruption would mask the exon as an intron, which would not be included in the final mRNA product; (ii) exon deletion involves the complete deletion of a single or multiple exons from the genome. Exon(s) within the range of two targeting sgRNAs would be excised. Mono-exon deletions could be designed for each *dystrophin gene* mutation type. For a multiexon deletion strategy, exons 45–55 (or exons 44–54) are deleted and could be applied to up to 60% of DMD patients, although this results in the production of a much smaller dystrophin protein as seen in Becker muscular dystrophy; (iii) another approach to avoid premature stop codons and recover the ORF is by inserting or deleting bases and making frame shifts instead of an exon deletion. NHEJ-mediated insertions or deletions may induce frame shifts and recover the ORF; (iv) *dystrophin* gene deletion mutations involving one or multiple exons could be rescued by a knock-in strategy of the deleted exon(s) to completely restore full length dystrophin protein expression. In this strategy, a donor template should be delivered in addition to Cas9 and sgRNA.

There are mainly four approaches that have been demonstrated to restore the open reading frame of dystrophin transcripts by genomic editing with CRISPR Cas9: (i) exon skipping by splicing acceptor disruption, (ii) exon deletion, (iii) NHEJ mediated frame shift, and (iv) exogenous exon knock-in [46] (Figure 1). The use of each approach can be catered to the type of DMD mutation to be targeted (Table 2).

## 7. Exon Skipping

Similar to the AON strategy mentioned above, CRISPR-Cas9-mediated mutagenesis of splicing acceptor (SA) sites could induce exon skipping to permanently restore the *DMD* ORF. As an example, a DMD patient lacking exons 48–50 (Δ48–50 DMD) fails to express the protein when exon 47 is followed by exon 51. However, if exon 47 is followed by exon 52, then the ORF can be restored (Figure 1()). Ousterout et al. demonstrated that this could be accomplished by removing a splicing acceptor (SA) in front of exon 51 by a pair of ZFNs in immortalized myoblasts from a DMD patient [47]. Similarly, another group reported the SA disruption of exon 51 in myoblasts derived from Δ48–50 and Δ45–52 DMD patients by CRISPR SpCas9 to restore dystrophin protein expression with a combination of TALEN and CRISPR SpCas9 strategies [48, 49]. Our group applied this approach to disrupt the SA of exon 45 in iPSCs derived from a Δ44 DMD patient using CRISPR SpCas9 and TALENs to skip exon 45 and successfully restore dystrophin protein expression after iPSCs were differentiated into myoblasts [13].

## 8. Exon Deletion

An alternative for exon skipping is to excise one or more targeted exons. Using CRISPR SpCas9 with Δ48–50 DMD patient-derived myoblasts, Ousterout et al. and colleagues deleted exon 51 by targeting the flanking introns 50 and 51 [50]. The result was a similar phenotype to that observed with SA disruption. The authors also employed a larger exon deletion strategy where they designed sgRNAs against introns 44 and 55 to remove a 336 kb genomic region of the *dystrophin* gene. Adeno-associated virus (AAV) delivery of either multiplexed TALENs or CRISPR Cas9 into Δ48–50 and Δ45–52 DMD patient myoblasts could also remove exons 45–55. Furthermore, Young et al. used three DMD patient-derived iPSC lines (Δ46–51, Δ46–47, and duplicated exon 50) to demonstrate that two sgRNAs could remove exons 45–55 and a larger portion of the intronic region [12]. Up to a 725 kbp region of genomic DNA was removed using this approach, and dystrophin-positive fibers could be observed in differentiated skeletal muscle cells in vitro as well as in vivo in transplanted *mdx* mice [12]. Although the adverse effects by the large genomic deletion need to be determined, these results are important from a cost perspective because they indicate that up to 60% of DMD patients could be converted to a BMD genotype by the multiexon deletion approach [50].

Exon deletion has also been useful in vivo in *mdx* mice, which have a premature stop codon in exon 23 of the *dystrophin* gene. Deletion of mouse exon 23 by CRISPR Cas9 can restore the ORF, similar to the approaches conducted in human cells. Three groups reported the delivery of either SpCas9 or SaCas9 and sgRNAs targeting upstream and downstream of exon 23 by AAV into *mdx* mice in vivo. All three of these groups performed localized and systematic delivery of Cas9 and sgRNAs by intramuscular or intraperitoneal injection and not only successfully recovered dystrophin expression but improved muscle function as well [51–53].

## 9. Frame Shifting

The NHEJ pathway can be utilized to induce insertions and deletions for resetting an ORF containing a premature stop codon and restoring dystrophin protein expression [54]. Theoretically, there is a one third chance of this event occurring, meaning that the approach is not highly efficient. Nonetheless, we applied this strategy to a region harboring a premature stop codon in exon 45 in iPSCs derived from a Δ44 DMD patient and successfully restored dystrophin protein expression [13]. Frame shift restoration of dystrophin by inducing indels in exon 51 and exon 53 of myoblasts derived from Δ48–50 and Δ45–52 DMD patients, respectively, was also shown to be effective [49].

## 10. Exon Knock-In

Exon knock-in with a DNA donor template by HR after nuclease-induced DNA cleavage offers the ability to restore full-length dystrophin protein expression. In Δ44 DMD patient-derived iPSCs, we succeeded in knocking in exon 44 at the 5′ end of exon 45 to obtain full length dystrophin protein in differentiated myoblasts [13]. While this strategy is appealing because there would be a complete restoration of native dystrophin protein, the frequency of the HR pathway is low and antibiotic selection is normally required. HR does not take place in G1-arrested cells such as mature myofibers, so it would be much less efficient in vivo. Indeed, Bengtsson et al. used two AAV vectors to deliver SpCas9, multiplexed sgRNAs, and a donor template in an *mdx* mouse harboring a nonsense mutation in exon 53 and found that HR was successful in 0.18% of total genomes compared with editing occurring in 2.3% of total genomes. In other words, NHEJ accounted for 92% of the edited genomes [55]. In this context, a target specific integration approach, such as HITI (homology-independent targeted integration) [56], might be applicable to insert the missing exon(s) at the appropriate locus. In addition, there is a limitation to the length of the HR template DNA that can be used depending on the delivery approach being used, meaning that it would be challenging to apply to large deletion mutations for restoring the original genomic DNA form of dystrophin.

## 11. The Expanding CRISPR Toolbox for Alternative Options to Treat DMD Patients

Depending on the targeted *dystrophin* gene sequence, other type II CRISPR Cas9 nucleases which have been reported such as those from *Streptococcus thermophilus* [58], *Neisseria meningitis* [59], or type V CRISPR Cpf1 may be appropriate for use. Cpf1 is approximately the same size as SpCas9 but does not have a tracrRNA component and consists of only crRNA. It has a PAM that is more applicable for T rich regions (i.e. “TTTN”) at the 5′ side of the targeted sequence. Cpf1 has distinct cleavage patterns compared with SpCas9 because it induces a staggered DSB as opposed to a blunt DSB. Insertion mutations and single base deletions were rarely observed with Cpf1, but out-of-frame mutation frequencies were comparable with SpCas9 [60].

Recently, new nucleases have been identified which may also be applicable for editing the *dystrophin* gene in the future. *Natronobacterium gregoryi* Argonaute (NgAgo) was reported to induce DSB guided by phosphorylated single-stranded DNA without the requirement of a PAM sequence, despite the fact that mammalian Argonaute normally processes RNA guided by an RNA (siRNA) template [61]. Although the reproducibility of the published results is still being debated [62], the potential to use other programmable nuclease systems would greatly expand the targeting range of candidate *DMD* mutations. Furthermore, through metagenomics of DNA extracted from bacteria and archaea that cannot be typically cultivated in the laboratory, new Cas9 nucleases, named CasX and CasY, have been recognized as smaller than SpCas9 and have unique sequence recognition for PAMs [63]. Continued exploration of these untapped sources for novel Cas9 nucleases may yield additional powerful tools for genome editing.

## 12. Alternatives to Genome Cleavage

Alternative approaches to treat DMD patients that do not rely on DNA cleavage could involve the utilization of catalytically dead Cas9 (dCas9) proteins fused with effector molecules for transcriptional activation, transcriptional inhibition, or specific base editing.

**Figure 2:**
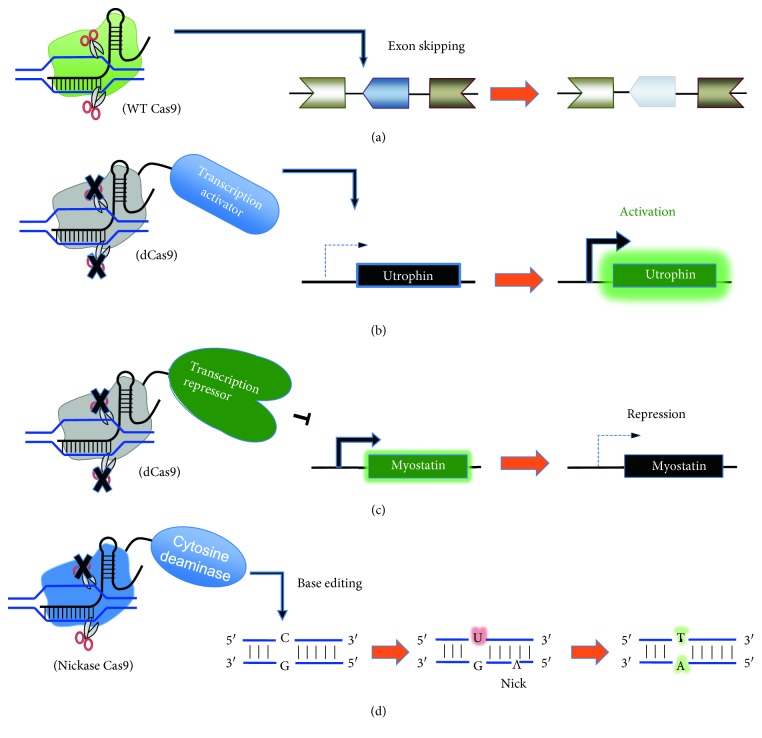
DSB and non-DSB mediated therapeutic approaches to potentially treat DMD. (a) WT Cas9 nuclease can be used to cleave DNA for exon skipping, frame shifting, or exon deletion as mentioned in Figure 1. (b) Catalytically inactive dCas9 fused with a transcription repressor such as KRAB can work as a sequence-dependent transcription repressor for a target gene such as *myostatin* to attenuate muscle wasting. (c) dCas9 fused with a transcription activator such as VP64 or p65 can work as a sequence-dependent transcription activator, in this case for activating *utrophin* expression to compensate for the absence of dystrophin. (d) Nickase Cas9 fused with a cytosine deaminase (i.e. APOBEC1 or AID homologue) can revert C to T by cytosine deamination. This can be used for correcting T → C mutations, or to disrupt premature stop codons or splicing acceptor sequences to induce exon skipping.

Utrophin (*UTRN*) is a paralog of dystrophin that is structurally similar and may be a candidate for transcriptional upregulation. Previous reports have shown that the overexpression of utrophin cDNA reduces the pathology of muscular dystrophy in *mdx* mice when overexpressed as a transgene [64]. Currently, phase I studies are being conducted with a drug molecule, called SMT 1100, to increase utrophin expression [65]. To transcriptionally activate a gene using dCas9, several groups have fused it with transcriptional activators such as VP64 (four tandem repeats of VP16) and p65 [66, 67]. Wojtal et al. recently reported that dCas9 fused with VP160 (ten tandem repeats of VP16) could boost utrophin protein expression in DMD patient myoblasts nearly 7-fold depending on the targeted promoter [68]. Thus, this technology is able to increase utrophin expression and bypass the risks associated with genomic cleavage of a functional Cas9 nuclease (Figure 2). In this context, stable activation of the utrophin gene is critical, hence a combination with demethylation of CpG or alike should also be considered.

Fusion of dCas9 with transcriptional repressor Kruppel-associated box domain (KRAB) has been shown to effectively inhibit the transcription of endogenous loci through the recruitment of heterochromatin [69]. One potential target of dCas9-KRAB in DMD patients is *Myostatin (MSTN)*, which is a cytokine released specifically by skeletal muscles cells that causes muscle atrophy [70]. One study showing the in vivo delivery of SaCas9 nuclease into wild-type mice targeting *MSTN* was able to attenuate muscle loss [71]. In a similar manner, dCas9-KRAB could be used to suppress Myostatin expression (Figure 2()); however, the effectiveness of dCas9-KRAB will depend on the chromatin status of the target DNA of the sgRNA, which was shown in cancer cells to cause a variation ranging between 60 and 80% transcriptional repression [69]. Again, stable alteration of the epigenetic state to maintain transcriptional suppression after the elimination of dCas9-KRAB will be essential to successfully incorporate this type of method.

Recently, the development of a nucleotide-specific base editor consisting of rat cytidine deaminase APOBEC1 (apolipoprotein B mRNA editing enzyme, catalytic polypeptide-like) [72] and AID ortholog PmCDA1 from sea lamprey [73] fused with a nickase version of Cas9 or dCas9, respectively, was shown to be able to induce C→T mutations. While it is estimated that 10% of DMD patients have point mutations [20], the base editing approach would be applicable to patients with T→C mutations that could be reverted back by Cas9-APOBEC1. This strategy may also be used to disrupt premature stop codons or splicing acceptor sites for inducing exon skipping (Figure 2). Although these new non-DSB techniques need to be optimized further, they may open new possibilities towards DMD therapy.

## 13. Considerations for Direct Use of CRISPR Cas9 to Treat DMD Patients In Vivo

AAV is a small (about 22 nm diameter) parvorvirus with a genome size of 4.7 kb and cannot replicate in the absence of a helper virus such as adenovirus or herpes simplex virus [74]. It has been engineered into a vector for transducing transgenes in vivo and has also been tested in gene therapy clinical trials for treating diseases such as hemophilia B and cystic fibrosis. For in vivo gene editing with the CRISPR Cas9 system, several groups have developed AAV vectors for the delivery of either Sp- or SaCas9 and sgRNA to treat DMD. These constructs were tested in *mdx* mice and could partially restore dystrophin expression either through local or systemic injection in skeletal muscle and cardiac cells [51–53]. However, when considering their use in human patients, AAV has several disadvantages. Up to 73% of adults have preexisting antibodies against different AAV serotypes from exposure to naturally occurring AAV infection during childhood [75], which is important because the anti-AAV antibodies could neutralize the viral particles before cell penetration and Cas9 transduction. The second disadvantage of AAV is that transgenes are constitutively expressed over a long period of time, especially in nondividing cells, which in the case of Cas9, may increase the probability of off-target cleavage. The third disadvantage is that the restrictive packaging capacity of the AAV capsid prevents both the Cas9 nuclease and sgRNA to be contained within the same vector. Indeed, for all three in vivo studies testing the effectiveness of the CRISPR Cas9 system, the nuclease and sgRNA were packaged into separate vectors [51–53]. Ideally, an all-in-one approach is desirable to increase in vivo delivery efficiency and to decrease the production cost.

The possibility of nonspecific DNA cleavage by CRISPR-Cas9 is a major concern in the field of gene editing [76] since it was reported by several groups that a high number of off-target events could occur depending on the uniqueness of the gene target sequence [77–79]. Off-target mutagenesis by ZFN nucleases has been associated with cellular toxicities [80]. Although numerous engineering approaches are being explored to minimize off-target cleavage by optimizing sgRNA structure and length or by adding regulatory elements to SpCas9 to limit nuclease activity, evaluation of the frequency of these events will be critical for therapeutic applications. Various evaluation methods exist, but the most sensitive only detects mutations at 0.1% frequency in in vitro cells, not in in vivo tissues [81]. Thus, the detection threshold may not be high enough to pick up rare off-target cleavage events, such as one in millions of cells. However, it should be noted that to date, no off target mutations for dystrophin sgRNAs have been reported in vitro or in vivo using CRISPR SpCas9 in pluripotent stem cells [12, 13, 51–53].

Another point of concern regarding the use of gene editing with CRISPR Cas9 in vivo is the immune response to the expressed Cas9 nuclease or corrected dystrophin protein. It is possible that newly expressed Cas9 nuclease and dystrophin may elicit an immune response. Indeed, it has been recently reported that Cas9 nuclease delivered into Ai9 or C57BL/6 mice by naked DNA electroporation or an AAV vector triggered an increase in the number of Cas9-reactive T cells [82]. Furthermore, the delivery of microdystrophin by AAV in a clinical trial failed to establish dystrophin protein expression but preexisting or de novo dystrophin protein reactive T cells were detected, suggesting that the transplanted cells were eliminated by immune clearance [24]. There is also evidence for an age-related increase in dystrophin reactive T-cells in DMD patients [83]. While these findings suggest that the immune system may be another obstacle to overcome in order to establish permanent treatment, limiting the expression of the Cas9 nuclease specifically to muscle cells utilizing a muscle-specific promoter may help to attenuate an immune response [55].

The way we envision the use of iPSCs going forward is to develop a screening platform to validate sgRNAs against the human *dystrophin* gene. Because each sgRNA has a variable activity and its own associated off target frequency, it is more relevant to test sgRNAs against the *dystrophin* gene in a human genome context, meaning that *mdx* mice are less informative for assessing specificity. To this end, patient-specific iPSCs could be used to test sgRNA activity and specificity directly in cells relevant to patients.

## 14. Conclusion

Much work has been done on editing strategies with CRISPR Cas9 to restore dystrophin expression in cells derived from DMD patients. Further discoveries of orthogonal CRISPR systems or other programmable nucleases will broaden our ability to precisely target the *dystrophin* gene. Advances on non-DSB type base editors or transcriptional regulator-based epigenetic editors might open new therapeutic approaches. Specificity and off-target effects are the biggest concerns regarding the safety of any programmable sequence-specific editors. In this context, DMD patient-derived iPSCs not only provide a disease-relevant context for validating a novel therapeutic approach but could also serve as an abundant source for testing specificity in a human genome context. Although most of the technologies mentioned here are years away from being used in patients, they provide exciting options for DMD treatment in future.

## Figures and Tables

**Table 1:** Reported DMD-iPSC lines and genotypes.

Patient information (sex/age/cell type)	iPSC reprogramming method	Mutation description	Ref.
Male/6 YR/fibroblast (Coriell ID: GM04981)	Multiple lentiviral vectors	ΔExons 45–53	[8]
Male/28 YR/fibroblast (Coriell ID: GM05089)	Multiple lentiviral vectors	ΔExons 3–5	[8]
Male/9 YR/fibroblast (Coriell ID: GM05169)	Multiple retroviral vectors	ΔExons 4–43	[11]
Male/3 YR/fibroblast	Multiple retroviral vectors	ΔExon 44	[10]
Male/9 YR/fibroblast	Multiple retroviral vectors	ΔExons 46-47	[10]
Male/9 YR/fibroblast (Coriell ID: GM05169)	Multiple sendai virus vectors	ΔExons 4–43	[9]
Male/10 YR/fibroblast (Coriell ID: GM03783)	Multiple sendai virus vectors	ΔExons 3–17	[9]
Male/23 YR/fibroblast (Coriell ID: GM04327)	Multiple sendai virus vectors	Exons 5–7 duplication	[9]
Male/18 YR/fibroblast (Coriell ID: GM05127)	Multiple sendai virus vectors	DNA 5533 G→T (protein E→X)	[9]
Male/11 YR/fibroblast (Coriell ID: GM03781)	Multiple sendai virus vectors	ΔExons 3–17	[9]
Male/NA/fibroblast	Polycistronic lentivirus vector	ΔExons 46–51	[12]
Male/NA/fibroblast	Polycistronic lentivirus vector	ΔExons 46-47	[12]
Male/NA/fibroblast	Polycistronic lentivirus vector	Exon 50 duplication	[12]
Male/3 YR/fibroblast	Multiple episomal vectors	ΔExon 44	[13]
Male/31 YR/T lymphocytes	Multiple sendai virus vectors	ΔExons 48–54	[14]
Male/13 YR/T lymphocytes	Multiple sendai virus vectors	ΔExons 46-47	[14]
Male/18 YR/fibroblast	Multiple lentiviral vectors	ΔExons 48–50	[15]
Male/14 YR/fibroblast	Multiple lentiviral vectors	ΔExons 47–50	[15]
Male/13 YR/fibroblast	Multiple lentiviral vectors	DNA 3217 G→C (protein E→X)	[15]
Male/10 YR/fibroblast	Multiple lentiviral vectors	ΔExons 45–52	[15]
Male/10 YR/fibroblast	Multiple lentiviral vectors	DNA 10171 C→T (protein R→X)	[15]
Male/8 YR/fibroblast	Multiple lentiviral vectors	DNA 4918-4919 ΔTG	[15]
Male/20 YR/fibroblast	Multiple lentiviral vectors	DNA 7437 G→A (protein W→X)	[15]

NA: not available; YR: years old.

**Table 2:** Summary of papers utilizing various CRISPR-Cas9 strategies to target DMD mutations in patient-derived cells.

Strategy	Target cell	DMD type	Genome editing target	Deletion size	Ref.
Mono or multiexon deletion	Myoblast	ΔEx48–50	Ex51 Ex45–55	336 kbp	[50]
Multiexon deletion	iPSC	ΔEx46–51 ΔEx46-47 Dup ex50	Ex45–55	530 kbp 670 kbp 725 kbp	[12]
Exon deletion	Myoblast	ΔEx48–50 ΔEx45–52	Ex51 Ex44–54 Ex53		[49]
Exon deletion	*mdx* mice (in vivo)	Nonsense mut in Ex23	Ex23	~0.5 kbp	[52]
Exon deletion	*mdx* mice (in vivo)	Nonsense mut in Ex23	Ex23	~1.2 kbp	[51]
Exon deletion	*mdx* mice (in vivo)	Nonsense mut in Ex23	Ex23	~0.3 kbp	[53]
Exon skipping	iPSC	ΔEx44	Ex45	18 bp	[13]
Exon skipping	Myoblast	ΔEx48–50 ΔEx45–52	Ex51 Ex53		[49]
Frame shifting	iPSC	ΔEx44	Ex45	2 bp insertion	[13]
Frame shifting	Myoblast	ΔEx48–50 ΔEx45–52	Ex51 Ex53		[49]
Frame shifting and exon deletion	Myoblast	ΔEx51–53	Ex50 Ex54	>160 kbp	[57]
Exon knock-in	iPSC	ΔEx44	Ex45		[13]
Exon knock-in and exon deletion	*mdx* mice (in vivo)	Nonsense mut in Ex53	Ex52-53 Ex53		[55]
